# Wild versus domestic prey in the diet of reintroduced tigers (*Panthera tigris*) in the livestock-dominated multiple-use forests of Panna Tiger Reserve, India

**DOI:** 10.1371/journal.pone.0174844

**Published:** 2017-04-05

**Authors:** S. S. Kolipaka, W. L. M. Tamis, M. van ‘t Zelfde, G. A. Persoon, H. H. de Iongh

**Affiliations:** 1 Institute of Environmental Sciences (CML), Leiden University, Leiden, The Netherlands; 2 Institute of Cultural Anthropology and Development Sociology (FSW), Leiden University, Leiden, The Netherlands; Université de Sherbrooke, CANADA

## Abstract

Grazing livestock in openly accessible areas is a common practice in the multiple-use forests of India; however, its compatibility with the reintroduction of tigers to these areas requires examination. Here, we investigated the diet of tigers in a livestock-dominated multiple-use buffer zone of the Panna Tiger Reserve, India. We hypothesised that the presence of feral cattle, along with open-access grazing practices in multiple-use forests, would increase the incidence of predation on livestock by tigers, even when wild prey are available. We used generalised linear models to test whether predation of livestock versus wild animals was influenced by (1) the sex and age class of tigers, (2) season, and (3) the distance of prey from the core-zone boundary of the reserve. Overall, sub-adult tigers and male tigers killed more livestock than wild prey, even when wild prey was available. In the winter and rainy seasons livestock were killed in higher numbers in the buffer zone than in summers, this may be because of the seasonally changing livestock herding patterns in the area. Further, with increasing distance from the core-zone boundary, all tigers killed more livestock, possibly because livestock were more easily accessible than wild prey. Our results show that open-access and unregulated livestock grazing is not currently compatible with large carnivore conservation in the same landscape. Such practices will lead to an increase in negative tiger-human-livestock interactions. In conclusion, we suggest the need to encourage locals to corral valuable cattle, leaving feral/unwanted livestock for tigers. This simple strategy would benefit both local inhabitants and tiger conservation in the multiple-use forests of India.

## 1. Introduction

The successful conservation of carnivores outside of protected areas is hindered by human–carnivore conflicts associated with livestock predation and attacks on humans [1]. In many countries, livestock provide stable livelihoods and sustenance for people [2]. However, when large carnivores inhabit the same landscapes that are also used by livestock, carnivores inevitably encounter and prey upon on livestock, as well as presenting a potential threat to people. Ultimately, the magnitude of livestock losses and ability of people to cope with such losses shapes their willingness to share the landscape with carnivores [3, 4]. Therefore, it is essential to minimize the incidence of predation where possible and ensure human safety to manage and successfully protect threatened carnivores in shared landscapes.

There are approximately 3000 tigers (*Panthera tigris*) left in the wild, and their numbers are still declining, despite sustained conservation efforts [1]. There are global efforts to safeguard the future survival of this iconic species in the wild. The forests of India support over 50% of the world's remaining wild tiger population; thus, these areas are important for the future survival of tigers. At present, wild tigers mostly inhabit protected tiger reserves in India, where human presence and activities are limited [5, 6]. However, to safeguard their future, networks of corridors have been proposed between tiger reserves, allowing free movement of tigers among protected areas, and access to a larger landscape with suitable habitat for population recovery [7].

India retains large tracts of government-controlled forests that extend beyond most protected tiger reserves. Within these forests, economically poor rural people pursue traditional livelihoods, collecting forest produce, such as fuelwood, fodder, timber, resins, fruits, and roots, in addition to grazing their cattle [8, 9]. For example, The per capita income of people living in the Panna district of Madhya Pradesh, India, where the Panna Tiger Reserve (PTR) is located is 523 US $ (or 31,389 Indian Rupees) [10]. The rural people living in the 42 villages in the buffer zone of the PTR keep approximately 25,000 cows, 5000 domestic buffalo and 15,000 goats [11]. Cows are mostly kept for sustenance and provide vital protein in the form of milk to rural residents. Buffalo can be purchased for 500 US $/individual (or 30,000 Indian Rupees) and reared for its high-fat milk, which is sold. Goats are bred for meat, and are valued at approximately 30 US $ (2000 Indian Rupees) for a 10 kg male goat. Local people have traditional rights to access multi-purpose forests, with their activities mostly controlled through informal community-level norms rather than regulated by formal government authorities [9]. However, the park management body has no control on how many cattle, whose cattle, or where the cattle are grazed in the buffer zone forests. Such unchecked grazing in multiple-use forests by local communities and poor regulation of forest use is widespread in India.

People’s use of forests can have both costs and benefit for carnivores using the same area [9, 12, 13, 14, 15]. For instance, poor livestock husbandry practices increase their vulnerability to predation [5, 12, 13, 16]. Furthermore, the unchecked and unregulated use of forests decreases the quantity and quality of habitat available for wildlife [17, 18] and local communities [18]. Consequently, a lack of regulations in shared landscapes makes it difficult to enforce sustainable natural resource management that also benefits wildlife [8]. On the other hand, people’s practices could also have positive benefits on carnivores. For instance, over 2,500 cattle perish each year from disease, predation, and seasonal extreme weather conditions in the 42 buffer zone villages of the PTR [11]. Villagers dump cattle carcasses at the village-forest fringes, where they are easily accessible as carrion for many carnivores [9]. Striped hyenas (*Hyaena hyaena*), village dogs (*Canis familiaris*), wild pigs (*Sus scrofa)*, jackals (*Canis aureus*), and raptors (e.g., vultures) opportunistically feed on these carcasses and thrive [9]. Large carnivores, like leopards, persist in highly modified farmland by killing available wild and domestic prey, like pigs and dogs [19]. Wolves (*Canis lupus*) also survive in the heavily degraded forests of central and western India by predating and scavenging on available wild and smaller domestic animals [20]. However, it remains unclear to what extent tigers exploit livestock in multiple-use zones, and to what extent tigers might be dependent on domestic animals to expand into habitat beyond the boundaries of protected areas [6]. Yet, such information is required because the Indian authorities plan to extend tiger conservation to create corridors beyond the reserves into these tracts of forests that are widely used by local communities.

The endangered tiger is a conservation dependent species [1]. From the perspective of recovering the tiger population, tigers need to expand beyond the confines of the protected reserves to maintain a strong gene pool and avoid local extinctions of the source population [21]. Therefore, both male and female tigers need to survive when outside protected reserves to facilitate population recovery. Here we investigate the case of tigers from PTR, where they became locally extinct during 2008 and were reintroduced into the same livestock dominated environment in 2009. The growing population of reintroduced tigers enter the adjoining multiple-use buffer zone where thousands of livestock graze, along with over 9000 feral cattle [9, 22, 23]. With this, the compensations paid for livestock losses by PTR management are also increasing (see the compensations records presented in (S3 Table). Tigers prey on large and intermediate bodied prey animals [24, 25, 26, 27, 28]. Thus, understanding how people and tigers interact in this landscape might provide conservation planners with important management insights for tiger conservation in livestock dominated landscapes. We hypothesised that the presence of feral cattle, along with open-access grazing practices in multiple-use forests, would increase the incidence of predation on livestock by tigers, even when wild prey are available. We examined kill data belonging to a group of radio-collared tigers inhabiting the PTR and we collected tiger scats in the multiple-use forest. We used generalised linear models to test the wild and domestic prey killed in relation to: (1) sex and age of tigers, (2) season, (3) the distance from the core-zone boundary of the reserve, and (4) water bodies. We expect our results to provide insights on livestock predation and management options to reduce tiger predation on livestock and facilitate the coexistence of people and tigers in multiple-use landscapes.

## 2. Methods

### Ethics statement

Field data collection permits were issued by the Chief Wildlife Wardens Office of Madhya Pradesh Forest Department, India to S. S. Kolipaka. Permit Number: 4029/9-6-2015. Data on tiger kills was obtained from the records of Panna Tiger Reserve, issued to S. S. Kolipaka under a mutual agreement. Permit Reference: Proceedings of the meeting 02.06.2014".

### Study area

**Fig 1:**
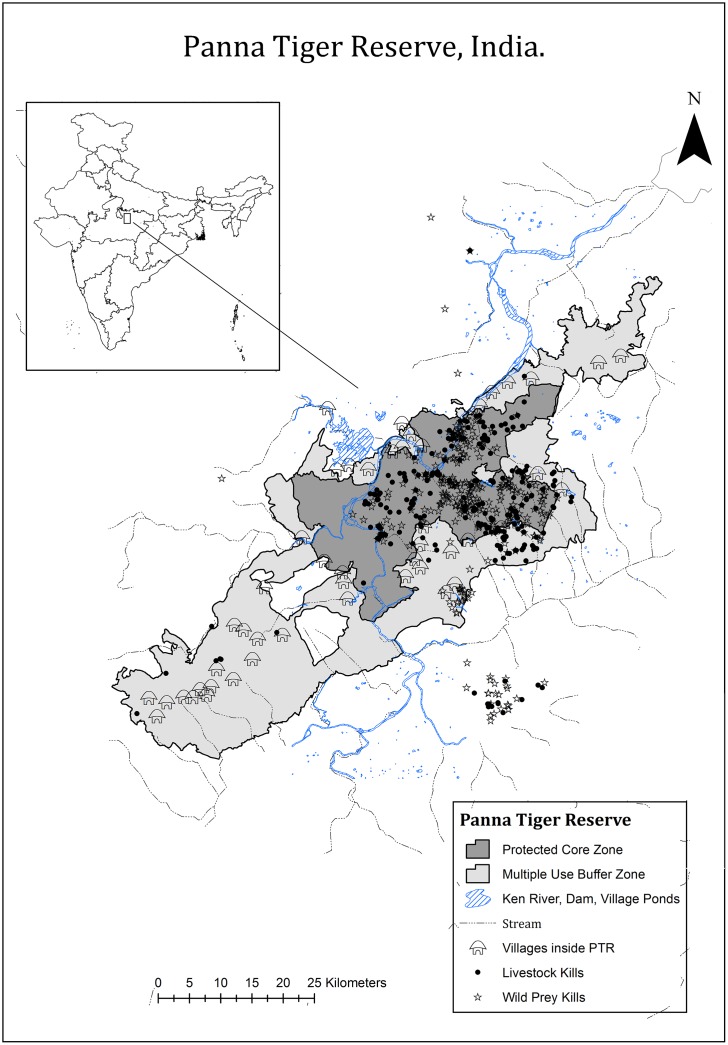
Map of the study area, the Panna Tiger Reserve. The core and multiple-use buffer zones, villages, and water bodies are shown. The circles and triangles represent the spatial location of wild and domestic prey items killed by 10 radio-collared tigers between 2009 and 2014.

Our study was carried out in the PTR (24° 274' N to 24° 905' N latitude; 79° 556' E to 80° 273' E longitude), which is a protected area that is located in the Bundelkhand region of north-central Madhya Pradesh, India. The reserve covers a 1645 km² area and is divided into two management units, a core zone and a multiple-use buffer zone (Fig 1). The core zone is 550 km², while the buffer zone is 1095 km². Human activity is restricted and natural resource extraction is prohibited in the core zone, whereas the buffer zone is a multiple-use zone. The tiger reserve is approximately 30 km at its widest (range: 10 to 30 km) and approximately 100 km in length (Fig 1), and is surrounded by multiple-use and human-dominated lands. Please see S1 Text for information on the climate, geography, and vegetation composition of the area.

### Traditional livestock management practices

Human presence and activity is high up to a 2 km distance from the centre of the villages during the daytime (07:00 to 17:00), with activity dropping between dusk to dawn (17:00 to 7:00). Water is a scarce resource in the study area, with reliable water bodies being limited. Consequently, people must share the same water bodies with their livestock and wildlife. Herders graze livestock up to a distance of 5 km from the village centre, with travel distances being highest during winter and rainy season to access good grazing sites and to keep cattle away from crops in the fields. This reverses in the summer months when temperatures get extremely hot and because livestock can also graze in fallow agricultural fields in villages. We examined the response of tigers using the buffer zones to these conditions near villages and water bodies.

### Distribution of livestock in the PTR

The presence and distribution of livestock in PTR are influenced by local husbandry strategies and prevailing cultural practices [9, 29]. Consequently, livestock that graze in the forests of the PTR buffer zone are grouped into three categories: (1) feral cattle, (2) native cattle that are owned but are not economically valuable, e.g., for commercial milk production (3) valuable buffalos and goats that are economically valuable, e.g., for milk and goats for meat. First, feral livestock primarily cows and bulls. Second, owned native cattle, lacking village grazing lands, villagers drive several thousands of cows to graze in the forests of PTR, mostly during the winter and monsoon farming seasons. Such cattle graze in the forests unaccompanied by herders during the day and aggregate near the village fringes at night. Most native varieties of cows do not yield sufficient milk; thus, they are not considered economically valuable by villagers. Yet people keep them because of religious sanctions that prohibit selling cows to tanners [9]. Third, valuable livestock that provide livelihoods for people, including milk yielding cows, and buffalos and goats. These valuable animals are herded during the day and are corralled at night.

### Study tigers

Over 20 terrestrial mammalian carnivores have been documented in the core and buffer zones of the PTR. Large terrestrial carnivores (>20 kg body weight) include the tiger, leopard, Indian wild dog or dhole (*Cuon alpinus*), wolf, striped hyena, sloth bear (*Melursus ursinus*), and domestic dog [22].

The tigers are part of a reintroduction project that commenced in 2009. Six founder tigers, which were reintroduced between 2009 and 2013 (5♀ and 1♂), and 4-second generation tigers (1♀, 3♂; born between 2010 and 2011). All 6 founder tigers and 6 of their offspring have been fitted with VHF radio collars by reserve authorities. Details of this equipment are provided by Sarkar et al. [23]. PTR tiger monitoring teams working in three 8-h shifts followed radio-collared tigers each day using a handheld VHF antenna between 2009 and 2014. The teams were tasked with recording the spatial locations of the tigers on an hourly basis. Following the signals from the transmitters, members from the monitoring team located individual tigers and homed-in. Tigers spent considerable time near carcasses and, whenever opportunity permitted, members from the team visually inspected kills after the animals left the carcass, recording details about the kill. Monitoring teams successfully recorded large bodied animal carcasses, but most of the carcasses of intermediate and smaller sized prey were either dragged deep into the thickets or were completely eaten by the tigers. Since we were more interested in livestock kills, the collected dataset provided sufficient information that was also reliable. The collected data were manually recorded into books maintained separately for each tiger and, where possible, photographs were taken. Recorded information on kills included the spatial locations of the kill, prey species, age group, and sex of prey. A small percentage (3%) of the kills could not be identified to the species level because carcasses were destroyed too much during the kill and subsequent feeding. Such information was excluded from the final analyses.

### Categorisation of tiger kills

We classified potential mammal prey into 3 size-based categories: large (>150 kg), intermediate (20–149 kg), and small (<19 kg). Potential large sized prey animals included the sambar deer (*Rusa unicolor*), nilgai antelope (*Boselaphus tragocamelus*), domestic cow (*Bos taurus*), and domestic water buffalo (*Bubalus bubalis*). Potential intermediate sized prey included the young of sambar deer, nilgai and cattle, chital deer (*Axis axis*), wild boar, chinkara antelope (*Gazella bennettii*), and four-horned antelope (*Tetracerus quadricornis*). Potential small-sized prey included the plains grey langur (*Semnopithecus entellus*), the domestic pig, goat, and domestic dogs [30].

### Analysis of scats

We collected tiger scats from the buffer zone during 2015 to investigate the presence of small prey that might be poorly represented by kill data. Since scats and kills were from different years, we did not include scats analysis here, but we did use the findings to support kill data as a validation technique. For details, see Table A in S2 Table.

### Statistical analysis

We considered depredation rates (domestic versus wild) in relation to 3 landscape characteristics: management zone (core versus buffer zones), within and beyond 2 km of villages, and within and beyond 250 m of water sources.

In the first analysis, prey (domestic or wild) was the dependent variable, while zone (buffer/core), generation (first generation = mature adults; second generation = young adults) and sex (male/female) of tigers, and season (summer, rainy, winter) were included as independent variables. In the second analysis, we included “Distance,” which was the distance from the core zone boundary to kill location and “near villages” (inside/outside 2 km of villages, which are high human density areas) as independent variables. In the third analysis, we used “water” and “near water” (inside/outside 250 m of water body) as the independent variables (rather than those of village).

All analyses were performed using generalised linear models (GLMs) in R 2.12.0 [31]. Adequate model fits were ensured by the stepwise removal of non-significant (significance p < 0.05) three-way and two-way interactions. We optimised the model based on all main effects and by only using the three–way and two-way interactions that were significant (See S1 Table for coefficients and the model selection procedure).

## 3. Results

### General diet

Our final analysis included 627 kills from 10 tigers (6 ♀ and 4 ♂) collected over a 5-year period between 2009 and 2014. Tigers primarily preyed on large and intermediate sized wild and domestic prey animals in the PTR (Table 1). Wild prey represented 54% of all kills made by tigers, while domestic prey animals represented 43%. Sambar deer represented 70% of all wild prey that were killed. Cows represented 87% of all domestic animals killed (Table 1). Small-sized prey animals (like reptiles, birds, and mongoose) represented <5% of the tiger diet, and were better represented in tiger scats compared to carcass counts; however, large prey were also predominant in scats. As a result, scat analysis was used only used to validate the presence and quantity of small prey in the tiger diet (see online supplement).

**Table 1:** Wild and domestic prey species killed by 10 radio-collared tigers in the core and the multiple-use buffer zones of the PTR between 2009 and 2014.

Prey Species	Prey Type	Average body weight of prey (Kg)	Body size category	Total Kills	% Kills	Kills in BZ	% Kills in BZ
**Cow (Feral + Domestic)**	D	150	L	243	37.56	86	51.8
**Sambar Deer**	W	136	L	245	37.87	52	31.3
**Nilgai**	W	182	L	49	7.57	15	9.0
**Domestic Buffalo**	D	150	L	35	5.41	0	0
**Sloth bear**	W	70	In	2	0.31	0	0
**Leopard**	W	45	In	3	0.46	0	0
**Cheetal deer**	W	47	In	21	3.25	1	0.6
**Wild Pig**	W/D	45	In	29	4.48	4	2.4
**Dog**	D	20	In	0	0	**	**
**Goat**	D	10	S	1	0.15	**	**
**Reptile**	W	2	S	0	0	**	**
**Bird**	W	2	S	1	0.15	**	**
**Mongoose**	W	1.5	S	0	0	**	**
**Unrecognised**		NA		18	2.78	8	4.8
**Total**				647	100.00	166	100.0

D = domestic; W = wild; L = large (>150 kg); In = intermediate (20–149 kg); S = small (<20 kg); BZ = buffer zone;

(**) = Represented by scats only and not kills (see supplement for more details); The average weight of the domestic cows and buffalos is less as they are native varieties and have a small structure compared to dairy cattle.

### Predation in relation to the core and buffer zone

**Fig 2:**
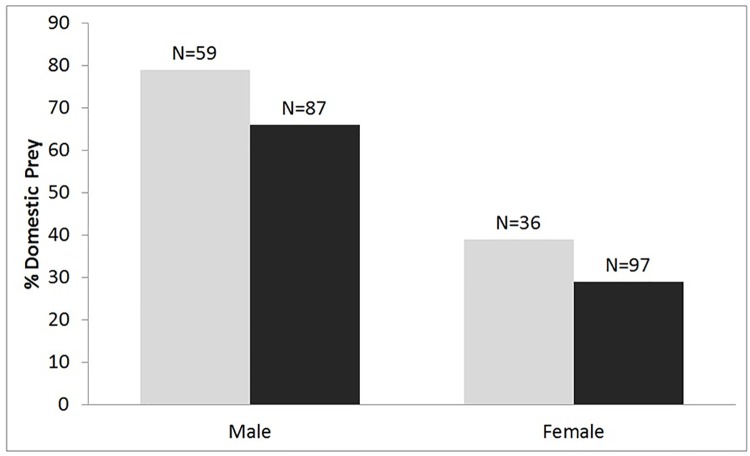
Percentage of domestic animals killed by male and female tigers in the core (black bars) and buffer (grey bars) zone of the PTR between 2009 and 2014. There was a significant interaction between sex * zone (df = 2, p < 0.05), with similar proportions of domestic animals being killed in core and buffer zones by males and females.

Domestic animals represented 57% of prey animals killed by tigers in the multiple-use buffer zone. In comparison, 40% of animals killed in the core zone were domestic. Male tigers killed a higher percentage of domestic animals in both the zones (66% and 79% in the core and buffer zone, respectively) than female tigers (29% and 39% in the core and buffer zone, respectively) (Fig 2; Table B in S2 Table). However, a similar proportion of male and female domestic prey was killed in the core and buffer zones (Fig 2).

**Fig 3:**
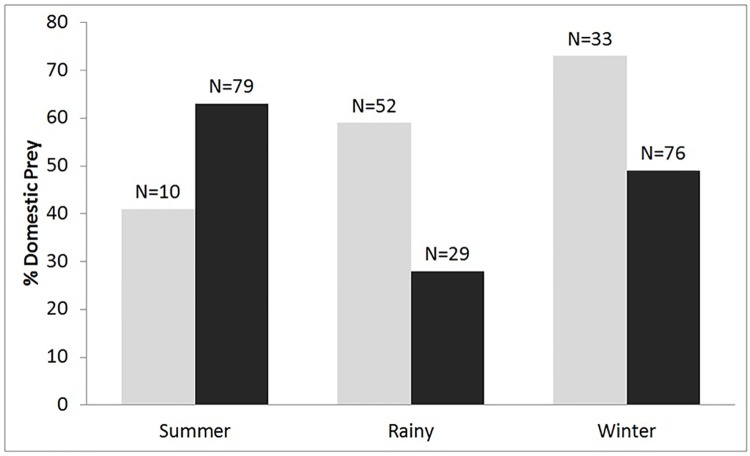
Percentage of domestic animals killed in the multiple-use buffer zone of the PTR by tigers during different seasons. There was a significant interaction between seasons * zone (p = < 0.001), with the proportion of domestic animals killed in the buffer being highest during the rainy season.

There was seasonal variation in the number of domestic prey animals killed by tigers in the core and buffer zones. In the core zone, tigers killed a higher percentage of domestic prey animals (66%) during the summer months, with this percentage dropping in the rainy and winter seasons. In the buffer zone, this situation was reversed, with tigers killing more domestic prey animals during the winter (73%) and rainy season (59%), and fewer in the summer (Fig 3).

### Predation incidents near areas of high human activity in the buffer zone

**Fig 4:**
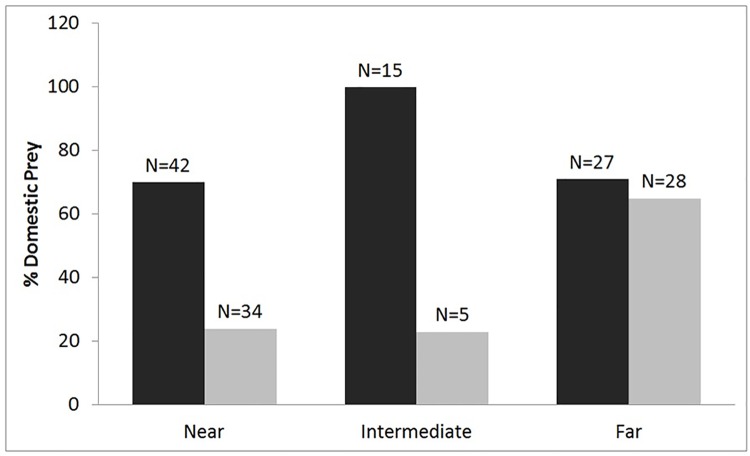
Percentage of domestic animals killed by male (black bars) and female (grey bars) tigers increased with distance from the core zone of PTR. At >10 km distance from the core zone, males and females killed domestic animals in similar proportions, with this result being statistically significant (df = 2, p = < 0.001).

Twenty-five percent of all kills made by tigers were within 2 km of villages. In these areas, tigers killed both domestic (52%) and wild animals (43%). However, proximity to villages was not statistically significant for predation, whereas distance from the core zone was significant (S1 Table). The predation of domestic animals increased with increasing distance from the core zone boundary to areas in the buffer zone (Table E in S2 Table). Male tigers killed more (N = 57) domestic animals than females (N = 39) up to a distance of 10 km from the core zone. At 10 km beyond the core zone, males and females killed similar proportions (Male (N = 27), Female (N = 28) of domestic animals (Fig 4).

**Fig 5:**
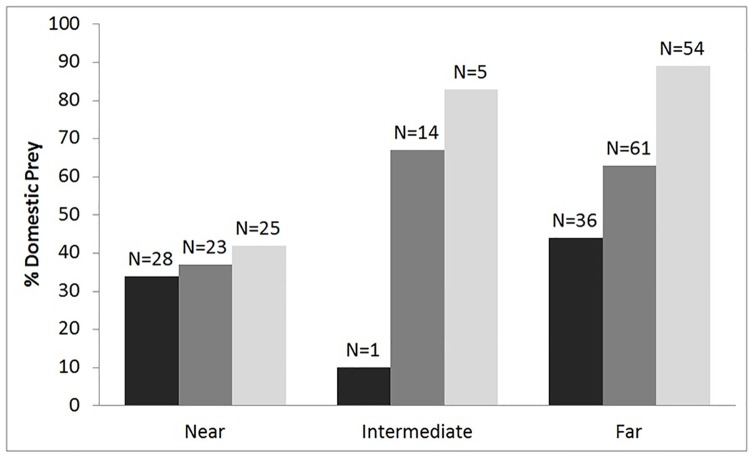
Percentage of domestic animals killed by tigers during the summer (black bars), rainy (dark grey bars), and winter (light grey bars) seasons with increasing distance from the core zone. The proportion of domestic animals killed doubled at >2 km distance from the core during the rainy and winter seasons (df = 4, p = 0.006), but remained low during the summer.

Tigers killed more domestic animals with increasing distance from the core zone during the rainy and winter seasons. The proportion of domestic animal kills in each season differed for each distance group from the core (Fig 5).

Twenty-nine percent of prey killed by tigers was near water (<250 m of water). Domestic and wild prey represented 45% and 52% of kills, respectively. However, there was no significant difference in the incidence of predation at <250 m and >250 of water bodies.

## 4. Discussion

This study demonstrated that tigers primarily fed on large-bodied prey, including both domestic and wild animals. Livestock were preferentially preyed upon with increasing distance from the protected core zone. Of note, female tigers primarily fed on wild prey when closer to the core zone, whereas male tigers targeted both domestic and wild prey. However, the proportion of domestic prey killed by males and females in core and buffer zones was comparable. Furthermore, seasonally changes in the distribution of domestic animals, reflecting seasonal variation in practices and livestock management, influenced the predation rate of livestock by tigers in the core and buffer zones. Our results demonstrate that open-access livestock grazing is not currently compatible with large carnivore conservation in the same landscape. However, our results also provide important information that could help reduce negative tiger-human-livestock interactions in the livestock-dominated buffer zone of the PTR.

### Prey items of tigers

Supporting previous studies, the tigers in the livestock-dominated PTR primarily preyed upon large and intermediate sized prey species [32, 24, and 25]. It is likely that our estimates based on kills alone underestimate the contribution of intermediate and small-sized prey, as demonstrated by our scat analyses (see supplementary material), and should be interpreted with caution. Our independent analysis of tiger scats validated that large animals represented the largest component of prey items; however, more small prey items were detected using this technique.

Sambar deer and cows represented the wild and domestic prey animals, respectively, that were primarily killed by tigers, supporting the results of previous studies [33, 26]. Unlike cows, buffalos tend to be accompanied by herders in our study area and are corralled at night [9]. The better herding and corralling practices extended to buffalos might explain the low losses of buffalo in comparison to cows. Our results show that the contribution of domestic animals to the diet of tigers was much higher in our study area compared to other geographically similar sites, where livestock is also predominantly found [34, 27]. However, similar levels of livestock predation have been detected for lions (*Panthera leo*) in similar livestock dominated habitats of western India [14]. The high predation of livestock by tigers in our study area is probably because of local livestock management practices. For instance, villagers follow a traditional practice called ‘Anna Pratha’. In this practice, villagers that cannot fend for their cattle during periods of stress (such as droughts) release their animals to fend for themselves or allow them to die out of sight [29]. As a result, thousands of feral cattle and free roaming cattle move inside the reserve area [22].

### Predation in relation to the protection zones

Overall, distance from the core zone boundary significantly explained the predation of domestic prey animals. More domestic animals were killed in the buffer zone than in the core zone of the PTR. The large number of wild animals killed near the core zone boundary was because of the availability of wild prey in these areas, and also because wild prey frequently raid crops in agricultural fields near peripheral areas [35].

Of note, more domestic animals were killed in the core zone during the summer, and vice versa during the rainy and winter seasons. Locally prevailing ecological conditions shape the movements of tigers [36] and livestock in the core and buffer zone, and probably influence predation rates. During the hot summer months, shepherds do not move their herds far from villages. They also allow livestock to graze on low-quality forage that is available in fallow agricultural fields. In comparison, feral and free roaming cattle move into the core zones to access better grazing and water sources. These movement patterns are reversed during the rainy and winter seasons.

Tigers, especially males, killed more domestic animals with increasing distance from the core zone boundary (up to 10 km), supporting that reported by Karanth and Sunquist [32]. In contrast, female tigers killed more wild prey animals than domestic prey animals in the core zone. Female tigers might preferentially target wild prey because they raise their young in the core zone and tend to have smaller home ranges than males. Thus, females probably choose areas that are far from human activity and where they are more likely to encounter wild prey. In contrast, males and young tigers move further afield and encounter more domestic prey.

### Predation in relation to villages and water bodies

Unexpectedly, predation near villages and water bodies in the multiple-use buffer zone was not significant. In other words, the incidence of tiger predation did not appear to increase near villages or water bodies, due to the presence of livestock. This result was interesting, especially because the presence of tigers was significantly high near villages but low near water bodies in the buffer zone, at least based on our unpublished data of the spatial movement patterns of tigers. However, the high presence of tigers did not translate into more killings. Our findings show that tigers spend considerable time near villages. It is likely that tigers are attracted to villages by people’s activities (such as dumping cattle carcasses near village fringes), un-corralled cows aggregating near villages at night, wild prey entering agricultural fields, and the presence of water bodies near village peripheries. However, as observed for lions in Kenya [37], tigers might not be able to fully utilise the available resources near villages or water bodies, where human presence and activity are high, while still continuing to use other parts of the human-dominated landscape. For instance, our study showed that livestock were preyed upon in greater numbers in other parts of the buffer zone and not necessarily near villages.

### Management suggestions

Our study showed that local practices, leading to the presence of livestock (feral and owned) throughout the multiple-use area, exhibit both costs and benefits to tigers using such areas, supporting the findings of Sharma et al. [15]. Livestock are important to people’s culture, livelihood, and well-being in India, as well as in most rural areas of the world. Consequently, livestock grazing will continue to remain a major land-use type in multiple-use landscapes, characterising such landscapes. Therefore, it is important to develop ways to decrease the predation of livestock by carnivores requiring conservation in multiple-use landscapes.

Our results clearly show that free-for-all livestock grazing is not compatible with large carnivore conservation in the same landscape. Such practices will cause negative tiger-human-livestock interactions to increase, particularly as the government is implementing initiatives to increase the size of the tiger population [38]. From the management perspective, this issue generates the need to consider new management options for tiger conservation in multiple-use landscapes. For instance, if the observed livestock management practices are widespread and commonly practised by thousands of people (like in the study area), attempting large-scale changes to people’s practices is not a viable option.

Instead, reserve management must innovatively model certain local practices to suit tiger conservation. For instance, as observed in the study area [9], many rural societies have traditional belief systems and norms that regulate their use and movement in forests. Likewise, people also have taboos towards hunting wild animals like nilgai and pigs that are potential prey of tigers [9]. These traditional practices reduce direct encounters between people and dangerous wildlife, including tigers, and also safeguard the prey of tigers [9]. By incorporating some of these traditional practices into management plans, managers might be able to retain existing levels of low interactions between people and tigers.

Furthermore, not all livestock predation incidents generate conflict with humans. Tigers might actually be providing a service to local communities by predating and regulating feral and unwanted animals. For instance, educational programs could be used to inform local communities about how tigers target prey items to encourage them to corral valuable cattle and leave feral/unwanted livestock for tigers. Local communities could establish ways to separate valuable village cattle from feral and unwanted cattle by means of tattoos or markings. In addition, fenced grazing zones could be set up for valuable cattle, restricting their movement in the forests. This simple strategy would benefit both local people and tiger conservation in the multiple-use forests of India, particularly in light of managers planning to implement corridors to connect protected areas to increase the gene flow among tiger populations.

## 5. Conclusions

This study provides novel insights into the prey choice of tigers in a human-dominated landscape with potential overlap between wild and domestic animal prey. While tigers were more likely to prey upon livestock with increasing distance from the core protection zone, we found no evidence that tigers kill more prey animals near villages or near shared water bodies. Thus, feral and free-roaming village cattle represent key targets for some tigers. In conclusion, for tigers to persist in multiple-use landscape, concepts that incorporate the needs of both wildlife and people must be implemented, rather than unregulated free-for-all land use.

## Supporting information

S1 Table Coefficients for analysis (1) Zone, (2) Village, (3) Prey age, (4) Prey Sex, (5) Water and (6) Scat and Kill. (DOCX)

S2 Table Details of data analysis. (DOCX)

S1 Fig Compensation amounts paid to livestock owners by PTR management between 2009 and 2014. (JPG)

S1 Text Climate, geography, vegetation and the practice of Anna Pratha in the study area. (DOCX)
