# Supplementary material for: Wild versus domestic prey in the diet of reintroduced tigers (Panthera tigris) in the livestock-dominated multiple-use forests of Panna Tiger Reserve, India
Source: PLoS One. 2017 Apr 5;12(4):e0174844. doi: 10.1371/journal.pone.0174844 (PMC5381891; doi:10.1371/journal.pone.0174844)
Supplement: S1 Table — (DOCX) [file pone.0174844.s001.docx]

**S1 Table: Coefficients for analysis (1) Zone, (2) Village, (3) Prey age, (4) Prey Sex, (5) Water and (6) Scat and Kill.**

**(1) Zone**

Response: Prey (Wild/Domestic)

Coefficients:

Estimate Std. Error z value Pr(>|z|)

(Intercept) 1.76420 0.50003 3.528 0.000418 ***

zone2 -1.52174 0.48181 -3.158 0.001587 **

sex2 -2.97532 0.56127 -5.301 1.15e-07 ***

gen2 -0.09017 0.35606 -0.253 0.800076

seasS -1.65467 0.53472 -3.094 0.001972 **

seasW 0.89834 0.48014 1.871 0.061344 .

zone2:sex2 1.28178 0.52350 2.448 0.014346 *

zone2:seasS 2.12970 0.60457 3.523 0.000427 ***

zone2:seasW -0.14699 0.56326 -0.261 0.794117

sex2:gen2 2.02289 0.52670 3.841 0.000123 ***

---

Signif. codes: 0 ‘***’ 0.001 ‘**’ 0.01 ‘*’ 0.05 ‘.’ 0.1 ‘ ’ 1

**(2) Village**

Response: Prey (Wild/Domestic)

Df Deviance Resid. Df Resid. Dev Pr(>Chi)

NULL 49 155.968

dist 2 22.686 47 133.282 1.185e-05 ***

sex 1 46.801 46 86.481 7.857e-12 ***

gen 1 8.629 45 77.852 0.0033079 **

seas 2 10.660 43 67.191 0.0048438 **

dist:sex 2 14.917 41 52.275 0.0005766 ***

dist:gen 2 12.794 39 39.480 0.0016664 **

dist:seas 4 14.310 35 25.171 0.0063700 **

---

Signif. codes: 0 ‘***’ 0.001 ‘**’ 0.01 ‘*’ 0.05 ‘.’ 0.1 ‘ ’ 1

**(3) Prey Sex**

Response: Prey sex

Df Deviance Resid. Df Resid. Dev Pr(>Chi)

NULL 39 61.974

Sex 1 10.5341 38 51.440 0.0011719 **

seas 2 6.1064 36 45.334 0.0472072 *

domwild 1 5.1283 35 40.205 0.0235382 *

sex:domwild 1 12.1229 34 28.083 0.0004981 ***

---

Signif. codes: 0 ‘***’ 0.001 ‘**’ 0.01 ‘*’ 0.05 ‘.’ 0.1 ‘ ’ 1

**(4) Prey Age group**

Response: Age group

Resid. Df Resid.Dev Df Deviance Pr(>Chi)

sex:gen:domwild 2 22 15.0122 -1 -5.4274 0.01982 *

sex:gen:zone 2 22 13.4381 -1 -3.8533 0.04965 *

sex:seas 2 23 18.9911 -2 -9.4063 0.009066 **

gen:seas 2 23 16.5089 -2 -6.9242 0.03136 *

domwild:seas 2 23 18.0732 -2 -8.4885 0.01435 *

Signif. codes: 0 ‘***’ 0.001 ‘**’ 0.01 ‘*’ 0.05 ‘.’ 0.1 ‘ ’ 1

**(5) Water**

Response: Prey (Wild/Domestic)

Df Deviance Resid. Df Resid. Dev Pr(>Chi)

NULL 43 194.605

Zone 1 14.094 42 180.511 0.0001739 ***

sex 1 80.130 41 100.381 < 2.2e-16 ***

gen 1 13.989 40 86.392 0.0001839 ***

seas 2 12.929 38 73.463 0.0015575 **

zone:sex 1 0.314 37 73.149 0.5754047

zone:gen 1 7.398 36 65.752 0.0065304 **

zone:seas 2 17.643 34 48.108 0.0001475 ***

sex:gen 1 10.172 33 37.936 0.0014259 **

sex:seas 2 1.701 31 36.235 0.4272070

gen:seas 2 0.714 29 35.521 0.6997409

zone:sex:seas 2 3.282 27 32.240 0.1938148

zone:gen:seas 2 6.494 25 25.745 0.0388828 *

Signif. codes: 0 ‘***’ 0.001 ‘**’ 0.01 ‘*’ 0.05 ‘.’ 0.1 ‘ ’ 1

**(6) Fisher exact test comparison scat and kill**

> x<-as.matrix(c(43,20,12,162,5,0))

> dim(x) <-c(3,2)

> x

[,1] [,2]

[1,] 43 162

[2,] 20 5

[3,] 12 0

> fisher.test(x)

Fisher's Exact Test for Count Data

data: x

p-value = 1.227e-14

alternative hypothesis: two. Sided
