# Supplementary material for: Wild versus domestic prey in the diet of reintroduced tigers (Panthera tigris) in the livestock-dominated multiple-use forests of Panna Tiger Reserve, India
Source: PLoS One. 2017 Apr 5;12(4):e0174844. doi: 10.1371/journal.pone.0174844 (PMC5381891; doi:10.1371/journal.pone.0174844)
Supplement: S2 Table — (DOCX) [file pone.0174844.s002.docx]

**S2 Table: Details of Data Analysis**

**Table A**: Percentage (%) of kills and frequency of occurrence (% FO) of prey items found in tiger scats showed the contributions of wild (W) and domestic (D) prey animals to the diet of tigers in the Panna Tiger Reserve. The 647 kills (2009 to 2013) and 56 scats (2015) were grouped into three size-based categories ((L) large, (In) intermediate, and (S) small) to demonstrate the relative contribution of prey size and prey type to the diet of the tiger.

|  | **Prey species and prey weight** | **Prey kills**  **(2009-2014)** | | **Frequency of Occurrence (FO) of**  **prey items**  **in scats**  **(2015)** | **(% FO)** |
| --- | --- | --- | --- | --- | --- |
| **Prey size category and type** | (Average body weights in kg) | Core + Buffer Zone | | Buffer Zone | |
|  |  | N | % | FO | % |
| **L – WP** | Sambar(136) | 245 | 37.9 | 14 | 17.5 |
| **L – WP** | Nilgai (182) | 49 | 7.6 | 7 | 8.8 |
| **L – WP** | Leopard (50) | 3 | 0.5 | ** | ** |
| **L – WP** | Sloth bear (70) | 2 | 0.3 | ** | ** |
| **L – WP** | Subtotal wild prey | 299 | 46.3 | 21 | 26.3 |
| **L – DP** | Cow (150) | 243 | 37.6 | 22 | 27.3 |
| **L – DP** | Buffalo(150) | 35 | 5.4 | ** | ** |
| **L – DP** | Subtotal domestic prey | 278 | 43.0 | 22 | 27.3 |
| **In- WP** | Chital (47) | 21 | 3.2 | 8 | 10.0 |
| **In- WP** | Pig (45) | 29 | 4.5 | 8 | 10.0 |
| **In- WP** | Subtotal wild prey | 50 | 7.7 | 16 | 20.0 |
| **In – DP** | Dog (20) | ** | ** | 2 | 2.5 |
| **In – DP** | Goat (10) | 1 | 0.2 | 2 | 2.5 |
| **In – DP** | Subtotal domestic prey | 1 | 0.2 | 4 | 5.0 |
| **S – WP** | Reptile (2) | ** | ** | 7 | 8.8 |
| **S – WP** | Bird (2) | 1 | 0.2 | 4 | 5.0 |
| **S – WP** | Mongoose (1.5) | ** | ** | 1 | 1.3 |
| **S – WP** | Subtotal wild prey | 1 | 0.2 | 12 | 15.1 |
|  | Unrecognised | 18 | 2.8 | 5 | 6.3 |
|  | Total | 647 | 100 | 80 | 100 |

Overall = Core zone + Buffer Zone;

Prey size: L = Large; In = Intermediate, and S = Small

Prey type: WP = Wild prey animals, DP = Domestic prey animals

Prey killed: (N) = Number of kills; (%) = Percentage contribution

Average body weight of an individual prey in kg (Sankar and Johnsingh, 2002)

FO = Frequency of Occurrence (% FO) = Percent of all scats containing each prey item

** = No values

**Table B**. Wild (w) and domestic (d) animals and the percentage of domestic (d%) animals killed by male and female tigers in the core and buffer zones of the Panna Tiger Reserve, India, between 2009 and 2013.

|  | Male tiger | | | Female tiger | | | Total | | |
| --- | --- | --- | --- | --- | --- | --- | --- | --- | --- |
| ZONE | w | d | d% | w | d | d% | W | d | d% |
| Buffer | 16 | 59 | 79 | 56 | 36 | 39 | 72 | 95 | 57 |
| Core | 44 | 87 | 66 | 232 | 97 | 29 | 276 | 184 | 40 |
| Total | 60 | 146 | 71 | 288 | 133 | 32 | 348 | 279 | 44 |

**Table C.** Wild (w) and domestic (d) animals and the percentage of domestic (d%) animals killed by tigers in the core and buffer zones during the summer (march to june), rainy (july to october), and winter(november to february) seasons in the Panna Tiger Reserve, India, between 2009 and 2013.

|  | Summer | | | Rainy | | | Winter | | | Total | | |
| --- | --- | --- | --- | --- | --- | --- | --- | --- | --- | --- | --- | --- |
| ZONE | w | d | d% | w | d | d% | W | d | d% | w | d | d% |
| Buffer | 24 | 10 | 41 | 36 | 52 | 59 | 12 | 33 | 73 | 72 | 95 | 52 |
| Core | 124 | 79 | 63 | 73 | 29 | 28 | 79 | 76 | 49 | 276 | 184 | 39 |
| Total | 148 | 89 | 38 | 109 | 81 | 43 | 91 | 109 | 55 | 348 | 279 | 44 |

**Table D.** Wild (w) and domestic (d) animals and the percentage of domestic (d%) animals killed by male and female tigers belonging to the first and second generations in the Panna Tiger Reserve, India, between 2009 and 2013.

|  | First generation | | | Second generation | | | Total | | |
| --- | --- | --- | --- | --- | --- | --- | --- | --- | --- |
| SEX | w | d | d% | w | d | d% | W | d | d% |
| Male | 21 | 50 | 70 | 39 | 96 | 71 | 60 | 146 | 71 |
| Female | 275 | 99 | 27 | 13 | 34 | 72 | 288 | 133 | 32 |
| Total | 296 | 149 | 33 | 52 | 130 | 71 | 348 | 279 | 44 |

First generation tigers are 6 founder tigers reintroduced between 2009 and 2013.

Second generation tigers are those that are born to the founder tigers starting from 2011.

**Table E:** Wild (w) and domestic (d) animals and the percentage of domestic (d%) animals killed by male and female tigers at different distances (near, intermediate, far) from the core zone of Panna Tiger Reserve, India, between 2009 and 2013.

|  | Near | | | Intermediate | | | Far | | | Total | | |
| --- | --- | --- | --- | --- | --- | --- | --- | --- | --- | --- | --- | --- |
| SEX | w | d | d% | w | d | d% | w | d | d% | w | d | d% |
| Male | 18 | 42 | 70 | 0 | 15 | 100 | 11 | 27 | 71 | 29 | 84 | 74 |
| Female | 109 | 34 | 24 | 17 | 5 | 23 | 15 | 28 | 65 | 141 | 67 | 33 |
| Total | 127 | 76 | 39 | 17 | 20 | 54 | 26 | 55 | 68 | 170 | 151 | 80 |

Near = Less than 2km; Intermediate = 2 to 10 km; Far= Over 10km

**Table F**: Wild (w) and domestic (d) animals and the percentage of domestic (d%) animals killed by first and second generation tigers at different distances (near, intermediate, and far) from the core zone of the Panna Tiger Reserve, India, between 2009 and 2013.

|  | Near | | | Intermediate | | | Far | | | Total | | |
| --- | --- | --- | --- | --- | --- | --- | --- | --- | --- | --- | --- | --- |
| GENERATION | w | d | d% | w | d | d% | w | d | d% | w | d | d% |
| First Gen | 105 | 47 | 31 | 17 | 4 | 19 | 13 | 7 | 35 | 135 | 58 | 30 |
| Second Gen | 22 | 29 | 57 | 0 | 16 | 100 | 13 | 48 | 79 | 35 | 93 | 73 |
| Total | 127 | 76 | 37 | 17 | 20 | 54 | 26 | 55 | 68 | 170 | 151 | 47 |

Near = Less than 2km; Intermediate = 2 to 10 km; Far= Over 10km

**Table G:** Wild (w) and domestic (d) animals and the percentage of domestic (d%) animals killed by tigers during the summer, rainy, and winter seasons at different distances (near, intermediate, far) from the core zone of the Panna Tiger Reserve, India, between 2009 and 2013.

|  | Near | | | Intermediate | | | Far | | | Total | | |
| --- | --- | --- | --- | --- | --- | --- | --- | --- | --- | --- | --- | --- |
| SEASON | w | d | d% | w | d | d% | w | d | d% | w | d | d% |
| Summer | 54 | 28 | 34 | 9 | 1 | 10 | 9 | 7 | 44 | 72 | 36 | 33 |
| Rainy | 39 | 23 | 37 | 7 | 14 | 67 | 14 | 24 | 63 | 60 | 61 | 50 |
| Winter | 34 | 25 | 42 | 1 | 5 | 83 | 3 | 24 | 89 | 38 | 54 | 59 |
| Total | 127 | 76 | 37 | 17 | 20 | 54 | 26 | 55 | 68 | 170 | 151 | 47 |

Near = Less than 2km; Intermediate = 2 to 10 km; Far= Over 10km

**Table H**. Male (MP) and female (FP) prey animals and percentage males (MP %) within wild and domestic prey animals, predated by male and female tigers in the Panna Tiger Reserve, India, between 2009 and 2013.

|  | Wild Prey | | | Domestic Prey | | | Total | | |
| --- | --- | --- | --- | --- | --- | --- | --- | --- | --- |
| SEX | MP | FP | MP% | MP | FP | MP% | MP | FP | MP% |
| Male | 43 | 5 | 89 | 73 | 53 | 58 | 116 | 58 | 67 |
| Female | 115 | 103 | 53 | 60 | 60 | 50 | 175 | 163 | 51 |
| Total | 158 | 108 | 59 | 133 | 113 | 54 | 291 | 221 | 57 |

**Table I**: Number of male (MP) and female (FP) prey animals and the percentage of male prey animals (MP%) predated on by tigers during the summer, rainy, and winter seasons in the Panna Tiger Reserve, India between 2009 and 2013.

| SEASONS | MP | FP | MP% |
| --- | --- | --- | --- |
| Summer | 120 | 75 | 62 |
| Rainy | 93 | 68 | 58 |
| Winter | 79 | 78 | 50 |
| Total | 292 | 221 | 57 |

**Table 10**. Young (Y) and adult (A) prey animals and the percentage of adult prey animals (A%) predated on by first and second generation male and female tigers in the core and buffer zones of the Panna Tiger Reserve, between 2009 and 2013.

|  |  | CORE | | | BUFFER | | | TOTAL | | |
| --- | --- | --- | --- | --- | --- | --- | --- | --- | --- | --- |
| GENERATION | SEX | Y | A | A% | Y | A | A% | Y | A | A% |
| First Gen | Male | 8 | 51 | 86 | 1 | 7 | 88 | 9 | 58 | 87 |
|  | Female | 27 | 246 | 90 | 4 | 50 | 93 | 31 | 296 | 91 |
| Second Gen | Male | 9 | 53 | 85 | 12 | 47 | 80 | 21 | 100 | 83 |
|  | Female | 1 | 15 | 94 | 9 | 19 | 68 | 10 | 34 | 77 |
| Total |  | 45 | 365 | 89 | 26 | 123 | 83 | 71 | 488 | 87 |

**Table 11**: Young prey animals (Y), adult prey animals (A) and the percentage of adult prey animals (A%) within domestic and wild prey animals that were predated on by first and second generation male and female tigers in the Panna Tiger Reserve, between 2009 and 2013.

|  |  | WILD | | | DOMESTIC | | | TOTAL | | |
| --- | --- | --- | --- | --- | --- | --- | --- | --- | --- | --- |
| GENERATION | SEX | Y | A | A% | Y | A | A% | Y | A | A% |
| First Gen | Male | 0 | 17 | 100 | 9 | 41 | 82 | 9 | 58 | 87 |
|  | Female | 12 | 216 | 95 | 19 | 80 | 82 | 31 | 296 | 91 |
| Second Gen | Male | 2 | 23 | 92 | 19 | 77 | 80 | 21 | 100 | 83 |
|  | Female | 0 | 10 | 100 | 10 | 24 | 71 | 10 | 34 | 77 |
| Total |  | 14 | 266 | 95 | 57 | 222 | 80 | 71 | 488 | 87 |

**Table 12**. Adult (A) and young (Y) prey animals and the percentage of adult (A%) prey animals from within wild and domestic prey that were predated on by tigers in the summer, rainy, and winter seasons in the Panna Tiger Reserve, India, between 2009 and 2013.

|  | Wild Prey | | | Domestic Prey | | | Total | | |
| --- | --- | --- | --- | --- | --- | --- | --- | --- | --- |
| SEASON | A | Y | A% | A | Y | A% | A | Y | A% |
| Summer | 120 | 1 | 99 | 75 | 14 | 84 | 195 | 15 | 93 |
| Rainy | 80 | 6 | 93 | 62 | 19 | 77 | 142 | 25 | 85 |
| Winter | 66 | 7 | 90 | 85 | 24 | 78 | 151 | 31 | 83 |
| Total | 266 | 14 | 95 | 222 | 57 | 80 | 488 | 71 | 87 |

**Table 13**. Adult (A) and young (Y) prey animals and the percentage of adult (A%) prey animals predated on by tigers in the core zone and buffer zones during the summer, rainy, and winter seasons in the Panna Tiger Reserve, India, between 2009 and 2013.

|  | Core Zone | | | Buffer Zone | | | Total | | |
| --- | --- | --- | --- | --- | --- | --- | --- | --- | --- |
| SEASON | A | Y | A% | A | Y | A% | A | Y | A% |
| Summer | 167 | 14 | 92 | 28 | 1 | 97 | 195 | 15 | 93 |
| Rainy | 81 | 9 | 90 | 61 | 16 | 79 | 142 | 25 | 85 |
| Winter | 117 | 22 | 84 | 9 | 9 | 79 | 151 | 31 | 83 |
| Total | 365 | 45 | 89 | 26 | 26 | 83 | 488 | 71 | 87 |

**Table 14**. Adult (A) and young (Y) prey animals and the percentage of adult (A%) animals predated on by male and female tigers during the summer, rainy, and winter seasons in the Panna Tiger Reserve, India, between 2009 and 2013.

|  | Male Tiger | | | Female Tiger | | | Total | | |
| --- | --- | --- | --- | --- | --- | --- | --- | --- | --- |
| Season | A | Y | A% | A | Y | A% | A | Y | A% |
| Summer | 56 | 5 | 92 | 139 | 10 | 93 | 195 | 15 | 93 |
| Rainy | 47 | 13 | 78 | 95 | 12 | 89 | 142 | 25 | 85 |
| Winter | 55 | 12 | 71 | 96 | 19 | 83 | 151 | 31 | 83 |
| Total | 158 | 30 | 84 | 330 | 41 | 89 | 488 | 71 | 87 |
