# Supplementary figures and images for: Wild versus domestic prey in the diet of reintroduced tigers (Panthera tigris) in the livestock-dominated multiple-use forests of Panna Tiger Reserve, India
Source: PLoS One. 2017 Apr 5;12(4):e0174844. doi: 10.1371/journal.pone.0174844 (PMC5381891; doi:10.1371/journal.pone.0174844)

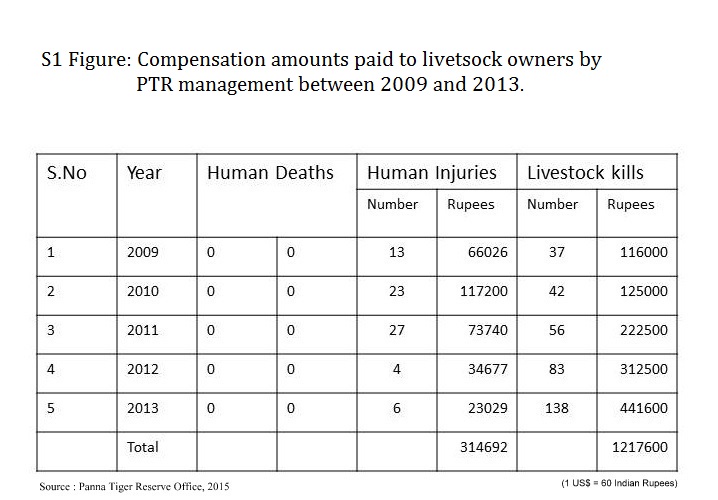

Supplement: S1 Fig — (JPG) [file pone.0174844.s003.jpg]
