# Supplementary material for: Wild versus domestic prey in the diet of reintroduced tigers (Panthera tigris) in the livestock-dominated multiple-use forests of Panna Tiger Reserve, India
Source: PLoS One. 2017 Apr 5;12(4):e0174844. doi: 10.1371/journal.pone.0174844 (PMC5381891; doi:10.1371/journal.pone.0174844)
Supplement: S1 Text — (DOCX) [file pone.0174844.s004.docx]

**S1 Text: Climate, geography, vegetation and the practice of Anna Pratha in the study area**

The study area has a tropical climate with three distinct seasons. The hot summer occurs between March and June, the wet season occurs from July to October, and the mild winter occurs from November to February. The daytime temperatures during the summer months exceed 45 °C, whereas the temperature drops to 3°C at night during winter. Annual rainfall is approximately 1100 mm, with the monsoon rains providing the principal source of water for large areas of the region [18]. Winter and summer showers are influenced by cyclones [9]. Rainwater from the hills flows through numerous streams and drains into the Ken River, the major waterbody in the area.

The terrain and vegetation in the reserve is hilly, with flat plateaus on the top and undulating plains on either side of the hills. Due to the hilly topography and quick drainage, the availability of surface water is a limiting factor throughout the entire area when the rains stop. Low water availability also shapes the vegetation in the area.

The reserve supports predominantly dry deciduous vegetation. On the plains, savannah grassland-woodland forests with short grasses and open thorny woodlands occur. In some areas, dry mixed and monotonous forest occurs. Bamboo grows mostly on the slopes.

**Over 12,000 cattle left to fend for themselves in parched Bundelkhand**

[**http://www.hindustantimes.com/india-news/over-12-000-cattle-left-to-fend-for-themselves-in-parched-bundelkhand/story-lIs0F2ACm5V4PSG1hoiHdI.html**](http://www.hindustantimes.com/india-news/over-12-000-cattle-left-to-fend-for-themselves-in-parched-bundelkhand/story-lIs0F2ACm5V4PSG1hoiHdI.html)

Jun 08, 2016 14:31 IST

[Neeraj Santoshi](http://www.hindustantimes.com/columns/neeraj-santoshi)
Hindustan Times, Bhopal


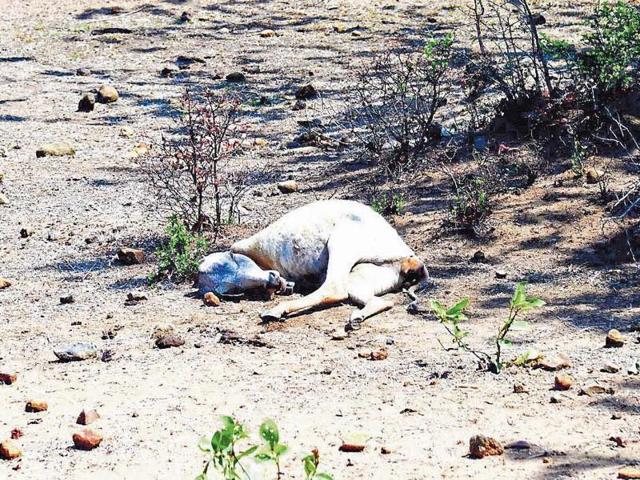


**In 37 villages, nearly 90% of the 14,000 cattle were abandoned by drought-affected villagers.(HT Photo)**

Unable to manage water and fodder for their cattle, scores of farmers in the drought-hit districts of Bundelkhand have released over 12,800 domestic animals this summer so far to fend for themselves, says a recent survey.

In the Bundelkhand region, it is a tradition called ‘Anna Pratha’ according to which people release their animals to fend for themselves or die away from their sight.

Hari Gaikwar, a farmer from Tikamgarh’s Tanga village, couldn’t bear to see his cows slowly dying of hunger and thirst before his own eyes. So he did the ‘Anna Pratha’ — like many others.

According to the survey conducted in 66 villages in three worst drought-hit districts of Bundelkhand — Tikamgarh, Sagar and Chhattarpur — there is no water left for cattle in 56% of the villages. In 37 villages, nearly 91% of the 14,000 cattle were released by helpless villagers. Hundreds of cattle have died, say the locals, while activists say the famine has already set in for the cattle.


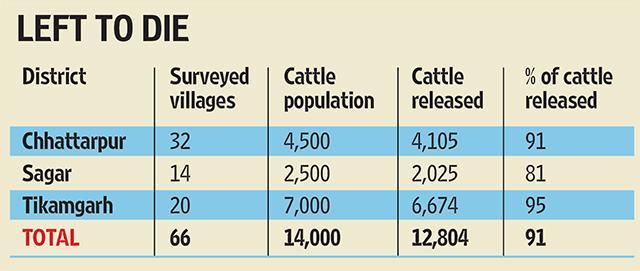


“In utter desperation, I released my five cows, two calves and one bull as I couldn’t arrange water or fodder for them. I couldn’t see them suffering before my own eyes. Only I know what it feels like when you see your cows leaving…”, said Gaikwar.

Sarika Sinha, regional manager of Action Aid MP, said the survey, which was conducted in April-May by Janpahal, a network of NGOs working in the drought-affected areas, Tikamgarh-based Madhya Pradesh Aapda Nivaran Manch and Action Aid, made them realise that famine-like conditions had already set in for domestic animals.

According to this survey, water was not available for cattle in 47% of the surveyed villages in Chhattarpur district, while in Tikamgarh district, the percentage of such villages was 45 %. Sagar district was the worst as 93% of the surveyed villages did not have water for the domestic animals.

Director animal husbandry Dr RK Rokde said he will ask his field staff in Bundelkhand to give him a report about the ground reality. “Many farmers in Bundelkhand release their cattle in summers. There is a tradition. But such a high figure is unusual. I will have to check with my staff. If the situation is alarming, we will take necessary steps,” he said.
